# Optimizing upside variability and antifragility in renewable energy system design

**DOI:** 10.1038/s41598-023-36379-8

**Published:** 2023-06-05

**Authors:** Diederik Coppitters, Francesco Contino

**Affiliations:** Institute of Mechanics, Materials and Civil Engineering (iMMC), Université catholique de Louvain (UCLouvain), Place du Levant, 2, 1348 Louvain-la-Neuve, Belgium

**Keywords:** Renewable energy, Energy science and technology, Engineering

## Abstract

Despite the considerable uncertainty in predicting critical parameters of renewable energy systems, the uncertainty during system design is often marginally addressed and consistently underestimated. Therefore, the resulting designs are fragile, with suboptimal performances when reality deviates significantly from the predicted scenarios. To address this limitation, we propose an antifragile design optimization framework that redefines the indicator to optimize variability and introduces an antifragility indicator. The variability is optimized by favoring upside potential and providing downside protection towards a minimum acceptable performance, while the skewness indicates (anti)fragility. An antifragile design primarily enhances positive outcomes when the uncertainty of the random environment exceeds initial estimations. Hence, it circumvents the issue of underestimating the uncertainty in the operating environment. We applied the methodology to the design of a wind turbine for a community, considering the Levelized Cost Of Electricity (LCOE) as the quantity of interest. The design with optimized variability proves beneficial in 81% of the possible scenarios when compared to the conventional robust design. The antifragile design flourishes (LCOE drops by up to 120%) when the real-world uncertainty is higher than initially estimated in this paper. In conclusion, the framework provides a valid metric for optimizing the variability and detects promising antifragile design alternatives.

## Introduction

The design of the energy system relies on the long-term forecasts of critical parameters such as resource availability (e.g., wind speed and solar irradiance), energy demand and energy prices. These forecasts generally carry significant uncertainty, as they fail to consider all pivotal events during the lifetime of the system^1^. To illustrate, the International Energy Agency (IEA) indicated forecasting errors up to 180% on the natural gas price and 25% on the energy demand in a retrospective review of their forecasts for 2020^2^. Therefore, considering these critical parameters as deterministic during decision-making will likely lead to suboptimal designs. Nonetheless, considering uncertainty in energy system decision-making processes is still an exception: Keirstead et al.^3^ highlighted that only 3 out of 219 reviewed studies on energy system models mentioned uncertainty or sensitivity analysis, out of which 2 performed an optimization under uncertainty. One of the main barriers to introducing optimization under uncertainty is the difficulty of characterizing the uncertainty, as the data on most parameters is limited (e.g., only a handful of predictions exists on the energy demand in the coming years)^4^.

Whenever uncertainty is considered in renewable energy systems, the uncertainty is mainly considered on the intermittent renewable energy supply (e.g., solar, wind), energy demand and energy carrier prices^5^. For intermittent renewable energy and demand, synthetic profiles can be constructed based on random instantaneous data sampled from distributions at every time step, which are characterized by statistical information derived from historical data, including mean, variance, skewness and kurtosis^6,7^. For parameters related to grid energy (e.g., electricity price, feed-in tariff, carbon tax), generally, only a handful of predictions are available. Therefore, discrete non-probabilistic approaches (e.g., interval analysis^8^, fuzzy set theory^9^) are most common, while normal and uniform distributions are typically assigned to these parameters whenever a probabilistic approach is considered^10^.

Once the uncertainties on the input parameters are defined, methods for optimization under uncertainty can be utilized to optimize an objective function while accounting for uncertainty in the input model parameters^11^. In the context of renewable energy systems, stochastic programming and worst-case scenario optimization are the most common approaches^12^. Stochastic programming considers a scenario-based approach to optimize the expectation of the system performance^13^. Alternatively, worst-case scenario optimization produces robust designs that can withstand the worst-case scenario derived from interval uncertainty on the input parameters^14,15^. This approach is highly dependent on the estimation of the worst-case scenario on the input parameters and generally leads to overconservative designs, as the designs are configured for a scenario with low probability^16,17^. As such, modifications to the algorithm were proposed to control the conservativeness of the robust counterpart^18,19^. Recently, an alternative to robust optimization, known as Robust Design Optimization (RDO), has gained attention in the context of renewable energy systems^20^. By considering probabilistic uncertainties on the model inputs, this method optimizes the expected performance and minimizes the variance of that performance^16^. The main advantages and disadvantages of the different methods are provided in Table 1.

**Table 1:** Overview of advantages and disadvantages of common optimization methods under uncertainty for renewable energy systems.

Method	Advantages	Disadvantages
Stochastic programming^12,13^	Considers entire variability of uncertain inputs	Requires a lot of data or assumptions on inputs
	Considers multiple scenarios which allows for flexibility	Large number of scenarios is computationally expensive
	Performs well under expected conditions	Complex algorithm
Worst-case scenario optimization^17,19^	Requires minimal data (only worst-case scenario)	Does not capture full range of uncertainties
	Easier to implement and interpret	Assumes that worst-case scenario can be predicted
	Solution that performs well under any scenario	Overconservative
Robust design optimization^16,20^	Considers entire variability of uncertain inputs	Requires a lot of data or assumptions on inputs
	Robust design is least-sensitive to uncertain environment	Computationally expensive
	Provides trade-off between robustness and performance	Robust design is often overconservative

Determining uncertainties for parameters in renewable energy systems is often hindered by the limited availability of data. Typically, only a small number of data points are available for a parameter, such as market data for investment costs of emerging technologies or discount rates for similar projects^10^. Moreover, the limited number of scenarios available for predicting the future evolution of a parameter can be unreliable. For instance, the Energy Information Administration’s (EIA) predictions for natural gas prices for electricity production between 1985 and 2015 were off by a factor of 3.32^1^, highlighting the potential inaccuracy of such predictions. Hence, there is often an insufficient amount of quality data to form a solid basis for constructing accurate distributions. Therefore, whenever uncertainty is considered on parameters with limited data, the data is often merged with expert judgement to characterize the distribution^21^. While experts can provide a good idea on the distribution type of a parameter (e.g., a lognormal distribution for the wholesale electricity price, as the price tends to increase in the next decade^22^), their estimation of the distribution parameters is typically poor, especially for higher-order moments (e.g., the variance) and skewed distributions^23^. Therefore, assuming that the compound uncertainty of the random environment, configured by the distributions on the input parameters, accurately represents the probability of each occurrence is relatively strong. For instance, there is a strong tendency to underestimate the uncertainty of predictions elicited from subject experts^24^. Moreover, representing these uncertainties by uniform distributions is inaccurate, especially for energy prices and demands, as these methods consider zero probability to outliers (i.e., tail events)^25^. Unbounded distributions, such as the normal distribution, capture tail events, but also, here, the probability is commonly underestimated for events away from the central position in the distribution^23^. This is because the probability of tail events is merely the result of an extrapolation on the distribution, defined by distribution parameters that are estimated from a set of predictions that are near the central position of the distribution, i.e., scenarios focus rather on the ordinary and the predictable than on the exceptions^26^. To illustrate, Shlyakhter et al.^25^ showed that, for the predictions on the primary energy demand of the USA considering the historical record of predictions, 13% of the scenarios deviate with at least 2.5 standard deviations. Instead, a normal distribution assigns a probability of about 1% for these scenarios to occur. Consequently, an underestimation of the compound uncertainty on the input parameters likely leads to an underestimation of the probability of events away from the central tendency of the distribution, thus an underestimation of critical events. Recently, in Europe, several industrial Combined Heat and Power units were taken out of operation following a tail event in their random environment: highly fluctuating electricity prices, high gas prices and overcapacity on the electricity market^27^. The 1.3 GW *Claus C* Combined Cycle Gas Turbine in the Netherlands, in operation since 2012, was shut down in 2014 because of a tail event in the market conditions (rising natural gas price and low electricity price on the market)^28^. Also, on a technical level, the probability of tail events is erroneous: Chen^29^ highlighted that the probability of power system blackouts is consistently and significantly underestimated. Overall, Maćkowiak and Wiederholt^30^ claimed that underestimating the probability of rare events leads to key decision-makers being unprepared to handle them, such as the global financial crisis, the European sovereign debt crisis, and the Fukushima nuclear accident.

**Figure 1:**
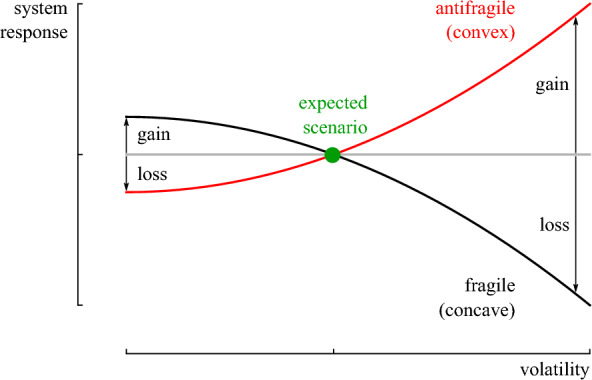
The response of a fragile and antifragile system when the input parameter deviates from the expected scenario. A fragile system response to variability results in limited gain but high losses. Instead, an antifragile system response results in limited losses but high gains.

While the probability of events away from the central tendency is inherently difficult to predict (with the black-swan event as a typical example of an unpredictable event^26^), it might not be necessary to know the probability of outliers to perform an effective optimization under uncertainty. Recently, Taleb^31^ introduced the concept of antifragility—the *true antonym* of fragility—which is a characteristic of systems that benefit from randomness and unexpected events in their quantity of interest. To illustrate, a fragile quantity of interest is the flight arrival time: variability in the environment is unlikely to reduce the flight time by more than a handful of minutes, while delays can build up to several days. In the opposite case, evolution is antifragile: A volatile environment eliminates vulnerable species that only flourish in specific conditions, while strong species evolve further^32^. To identify if a system is antifragile, Taleb^31^ argued that one should quantify the system’s response to outliers rather than trying to predict the probability of occurrence for these events. Following this heuristic, a fragile system has a concave response to variability. It ends up with more harm than profit, while an antifragile system has a convex response that results in more gain than harm (Fig. 1)^33^. Recently, de Bruijn et al.^34^ investigated this heuristic as a design criterion for dynamic systems and concluded that the antifragile version yields more favorable results in a random environment defined by substantial outliers.

In a framework where the random environment is defined by precise distributions, Taleb and Douady^35^ identified fragility as the increase in probability density of a system response below a certain performance threshold (i.e., the loss domain) when the uncertainty in the random environment increases. Similarly, antifragility corresponds to the increase in probability density above a certain performance threshold (i.e., the gain domain), combined with a robust tail below the performance threshold. Thus, when designing a system for a random environment that is likely underestimated, the resulting antifragile design has an increased probability of operating in the gain domain in the actual random environment than in the estimated random environment. Systems with this characteristic have a positively-skewed distribution on their response^36^. However, antifragility comes at the expense of reducing the performance near the central tendency, i.e., increasing the skewness of a distribution towards the gain domain lowers the performance of the most-probable events. To this extent, designing for antifragility is comparable with risk aversion in decision making^37^, where one accepts a lower expected return for increased security towards risk. Although, in antifragility, one accepts a lower expected return for increased security and potentially large gains. Recently, positively skewed returns gained interest in portfolio management^38^ and asset allocation^39^, where investors mentioned their preference for positive skewness above a higher mean and lower volatility.

In this paper, we propose two main novelties: promoting upside variability and detecting antifragility in the performance of renewable energy systems. Current optimization methods under uncertainty mainly focus on robustness, either by optimizing the performance under the worst-case scenario or by minimizing the sensitivity of the performance to input variability. While optimizing for robustness limits the effect of input uncertainty on the system performance, it neglects input variability that improves performance beyond the average in the most probable scenarios (i.e., upside variability). For common quantities of interest in renewable energy systems, such as efficiency, cost, and carbon intensity, upside variability is encouraged, as it would result in, e.g., higher efficiency, lower cost, or lower carbon emissions than in the most probable scenarios. Therefore, to favor upside variability on a quantity of interest (i.e., the first novelty), we introduce the Upside Potential Ratio (UPR) as an optimization objective, which protects against downside variation while promoting upside variability. In addition, existing methods consider the entire random operating environment or the worst-case scenario as perfectly known. However, the uncertainty on critical input parameters is based on only a handful of data point and is, therefore, often significantly underestimated for events away from expected conditions, leading to a random environment defined with low precision. Therefore, as a second novelty, we propose using skewness as an objective to represent antifragility in the quantity of interest. This antifragility metric overcomes the limitation of underestimating the random environment, as positively-skewed distributions on the quantity of interest mainly augment positive outcomes if the actual random environment is more uncertain than anticipated. In summary, the UPR and skewness are integrated as objectives in the Antifragile Design Optimization framework (ADO), which is a novel, multi-objective approach to optimize the central tendency, variability, and antifragility of renewable energy systems under uncertainty.

## Methods

The ADO framework provides a methodology to perform multi-objective optimization under uncertainty, considering the design’s central tendency, variability and antifragility. First, the characterization of the random environment is illustrated, followed by the Uncertainty Quantification (UQ) method to propagate the random environment through the system model and quantify the distribution of the quantity of interest. Once this distribution is defined, the objectives that represent the design’s performance in terms of central tendency, variability and antifragility are outlined. After that, the multi-objective optimization algorithm is described, optimizing these three objectives by controlling the design variables. This section concludes with an overview of the ADO method.

### Uncertainty characterization of the random environment

To characterize the uncertainty on the renewable energy system parameters, the data is generally rather limited and does not provide a solid basis for statistical inference^1^. In addition, subject experts typically provide poor estimates on distribution parameters, especially in the case of skewed distributions^23^. Therefore, we adopted a knowledge-based empirical approximation method based on the method described by Arnold and Yildiz^40^. In this method, expert opinions are elicited into quantiles and distribution types. Based on the quantiles and the distribution type, the distribution parameters are quantified using the method of Cook^41^.

**Figure 2:**
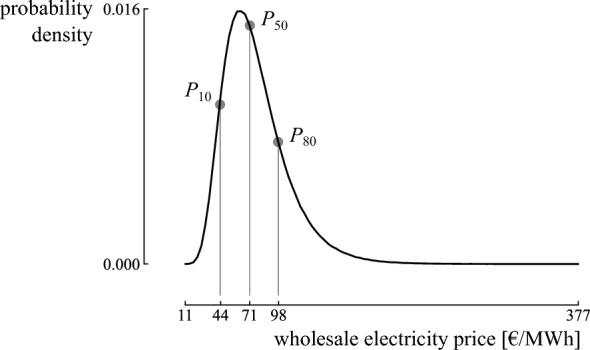
The characterized distribution on the wholesale electricity price, based on two quantiles: $P_{10} =$44 €/MWh and $P_{80} =$98 €/MWh. The median is $P_{50} =$71 €/MWh, which corresponds to the median of the adopted dataset.

To illustrate, the method is applied to the wholesale electricity price in Belgium. The Belgian electricity system operator (Elia) provided six scenarios for the wholesale electricity price, ranging between 44 €/MWh and 98 €/MWh^42^. Salimi et al.^22^ indicated that the evolution of the wholesale electricity price follows a lognormal distribution. The method starts from a normal distribution to retrieve the lognormal distribution parameters. For a normal distribution *X*, the mean ($\mu$) and standard deviation ($\sigma$) can be quantified for $x_1$ with quantile $p_1$, and $x_2$ with quantile $p_2$, as follows^41^:

1 $$\begin{aligned} \mu = \dfrac{x_1 F^{-1} \left( p_2 \right) - x_2 F^{-1} \left( p_1 \right) }{F^{-1} \left( p_2 \right) - F^{-1} \left( p_1 \right) }, \end{aligned}$$

2 $$\begin{aligned} \sigma = \dfrac{x_2 - x_1 }{F^{-1} \left( p_2 \right) - F^{-1} \left( p_1 \right) }, \end{aligned}$$

where *F* is the Cumulative Distribution Function (CDF) of the standard normal random variable *Z*, i.e., $X = \sigma Z + \mu$. Once the formula for the normal random variable is established, the lognormal distribution *Y* is found using $Y = {\log}X$, such that $P \left( Y < {\log} x_i \right) = p_i$. To equal the median of the scenarios with the median of the distribution, we assume the following quantiles on the worst case scenario and best case scenario, respectively: $P \left( Y < 44 \right) = 0.1$ and $P \left( Y < 98 \right) = 0.8$. This results in a lognormal distribution with a median of 71 €/MWh, which equals the median of the scenarios. The corresponding distribution parameters are $\mu = 4.267$ and $\sigma = 0.377$ (Fig. 2). We refer to Cook^41^ for the details on deriving the parameters for other distributions.

### Uncertainty quantification

A UQ method is required to propagate the input distributions through the system model and quantify the distribution on the quantity of interest. Crude Monte Carlo Simulation is typically adopted, randomly generating scenarios from the distributions on the input parameters until sufficient ($10^3{-}10^4$) values on the quantity of interest are collected to make a statistically valid distribution^43^. Despite being easy to implement, the method is computationally intractable in an optimization framework when the model takes more than a few seconds to calculate the result for a single scenario^44^. Therefore, we adopted the computationally-efficient PCE surrogate modelling technique^45^. We used the Python package RHEIA, developed in our research group, to construct the PCEs^46^. While a concise overview of the Polynomial Chaos Expansion (PCE) method is presented in the subsequent paragraph, a comprehensive exposition of its various stages is available in Sudret^45^.

A PCE model ($\hat{M}$) replicates the input-output relation of the system model (*M*) by generating a series of multivariate orthogonal polynomials ($\varvec{\Psi }$), weighted by coefficients (*u*):

3 $$\begin{aligned} \hat{M}(\varvec{\xi }) = \sum _{\varvec{\alpha } \in \textbf{N}^d} u_{\varvec{\alpha }} \varvec{\Psi }_{\varvec{\alpha }} (\varvec{\xi }) \approx M(\varvec{\xi }), \end{aligned}$$

where $\varvec{\xi } = (\xi _1,\xi _2,...,\xi _d)$ is a vector for the input distributions, *d* corresponds to the number of input distributions and $\varvec{\alpha }$ is a multi-index with *d* indices. In the multi-index of each multivariate polynomial, each index corresponds to the degree of a univariate polynomial. Hence, the multivariate polynomials are tensor products of univariate polynomials, and these univariate polynomials are selected in such a way that univariate polynomial $\Psi _i$ is orthogonal to input parameter distribution $\xi _i$. For well-known distributions, the corresponding orthogonal polynomial families are known^45^. To illustrate, the Legendre polynomial family corresponds to a uniform distribution.

When the PCE series is infinite, i.e., when multivariate polynomials up to an infinite degree are included in the PCE, the representation of the actual model is exact but computationally intractable^45^. Therefore, the PCE is truncated based on a conventional truncation scheme that limits the number of multivariate polynomials based on a limiting multivariate polynomial degree (*p*). As the sum of all indices in a multi-index correspond to the total degree of the multivariate polynomial, the multi-indices that comply with this limitation can be stored in $\mathcal {A}^{d,p}$:

4 $$\begin{aligned} \mathcal {A}^{d,p} = \left\{ \varvec{\alpha } \in \mathbb {N}^d : |\varvec{\alpha }| \le p \right\} . \end{aligned}$$

The amount of multi-indices complying with the limitation equals:

5 $$\begin{aligned} \text{card} \left( \mathcal {A}^{d,p} \right) = {p + d \atopwithdelims ()p} = \dfrac{\left( d + p \right) !}{d! p!} = P + 1. \end{aligned}$$

In this way, only the multivariate polynomials up to a degree *p* are added to the truncated series. To illustrate, for a system with two input uncertainties ($d=2$) and a maximum total degree of 3 ($p=3$), 10 multivariate polynomials are added to the PCE. The limiting total degree *p* is a user-defined constant and depends on the non-linearity of the input-output relation within the uncertain space defined by the input uncertainties. To quantify the corresponding coefficients using regression, $2(P+1)$ training samples are suggested to achieve a well-posed least-square minimization problem^45^. To generate the training samples, the quasi-random Sobol’ sampling technique has been adopted^47^. Finally, once the PCE surrogate model is constructed, the distribution on the quantity of interest is calculated using Monte Carlo Simulation ($10^6$ samples) on the PCE. As the PCE is an analytical function, this evaluation is nearly instantaneous on a common laptop.

Leave-One-Out (LOO) cross-validation is used to estimate the error of the Polynomial Chaos Expansion (PCE) surrogate model. In this method, first, a PCE is constructed without using the information from one of the training samples $\varvec{x}^{ \left( i \right) }$. The residual error is then computed on the left-out sample as the difference between the actual model result $M(\varvec{x}^{ \left( i \right) })$ and the PCE prediction $\hat{M}^{\backslash i}(\varvec{x}^{ \left( i \right) })$ without information on that point, i.e.,

6 $$\begin{aligned} \Delta _i = M \left( \varvec{x}^{ \left( i \right) } \right) - \hat{M}^{\backslash i} \left( \varvec{x}^{ \left( i \right) } \right) . \end{aligned}$$

This procedure is repeated for each training sample. The LOO error is defined as the sum of the squared residual errors over all samples and is normalized by the variance of the model outputs ($\text{Var} \left[ \varvec{y} \right]$). With a total of $2 \left( P+1 \right)$ training samples, the LOO error can be expressed as

7 $$\begin{aligned} E_\text{LOO} = \dfrac{1}{2 \left( P+1 \right) \text{Var} \left[ \varvec{y} \right] } \sum _{i=1}^{2 \left( P+1 \right) } \Delta _i^2. \end{aligned}$$

To simplify the computation of the LOO error for a single PCE, the expression can be written in terms of the diagonal terms $h_i$ in the least-square minimization matrix, as discussed in Sudret^45^. In conclusion, the limiting total degree *p* can be iteratively increased until a satisfying LOO error for the PCE is obtained.

### Objectives for central tendency, variability and antifragility

Once the distribution of the quantity of interest is defined with respect to the input distributions, the next step is to extract objective functions to quantify the performance of the stochastic quantity of interest. Three objectives represent the performance in central tendency, variability and antifragility, respectively.

The first objective is to optimize the central tendency of the distribution. A metric of central tendency aims to summarize the distribution with a single value by representing the central position. Hence, this value indicates the typical, expected performance. Standard measures of central tendency include the arithmetic mean, the median and the mode. As the distribution type for the quantity of interest is not known a priori—it depends on the input distributions and the non-linearity of the model—the choice of the metric to represent the central tendency is not straightforward. While the mean is adopted as an objective to optimize the central tendency in typical methods for optimization under uncertainty (e.g., RDO), the metric deviates from the central position when the distribution is skewed. In energy system optimization, skewed distributions in the random environment are expected (e.g., wholesale electricity price). In addition, energy system models are likely non-linear, especially when the quantity of interest is calculated over a long-term horizon (years) with high resolution (hours). Therefore, even when symmetric distributions are propagated through the model, skewed distributions can end up on the quantity of interest. Instead, the mode and median are robust towards outliers. The mode provides the most probable outcome, which is a valuable metric when a clear peak shapes the distribution. However, the mode is fragile towards a wide distribution with a small cluster of identical responses, which significantly deviates from a large cluster with slight differences in the response. Therefore, we consider the adoption of the median to represent the central tendency for the distribution of the quantity of interest.

The second objective optimises the variability of the quantity of interest. Typically, quantities of interest for energy systems are optimized on one side of the domain but softly constrained on the other side of the domain. To illustrate, the levelized cost of produced electricity for a power plant is minimized. Still, an upper limit exists from where the power plant is no longer economically viable to operate^27^. In a Haber-Bosch process, the hydrogen production in an electrolyzer is maximized, but a minimum amount of hydrogen production is required to sustain the reaction^48^. The CO$_2$-emissions for a country-scale energy system are minimized, but they should meet a CO$_2$ emission reduction target^49^. Therefore, the variability of the distribution should be considered such that the distribution has a limited probability of violating the minimum acceptable performance. Ideally, variability results in outcomes significantly better than a minimum acceptable performance, while the potential outcomes worse than a minimum acceptable performance are improbable and only slightly worse. These characteristics are summarized in the Upside Potential Ratio (UPR), a reward-risk ratio that considers skewed returns with respect to a minimum acceptable performance ($\Omega$)^50^:

8 $$\begin{aligned} \text{UPR} = \dfrac{\int _{\Omega }^{+ \infty } \left( x - \Omega \right) \text{Pr} \left( x \right) }{ \sqrt{ \int _{- \infty }^{\Omega } \left( x - \Omega \right) ^2 \text{Pr} \left( x \right) }}. \end{aligned}$$

**Figure 3:**
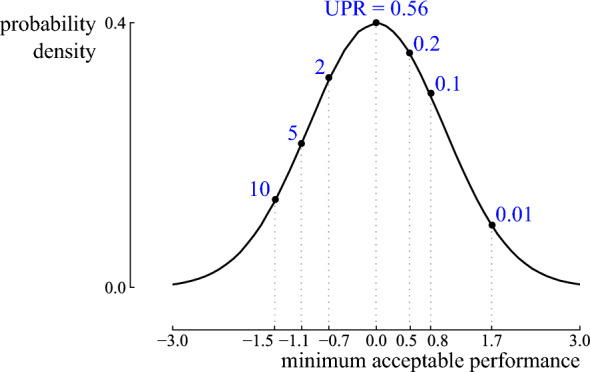
Upside Potential Ratio (UPR) for different minimum acceptable performances for a standard normal distribution. The lower the minimum acceptable performance, the higher the probability of attaining a performance higher than the minimum acceptable performance, which increases the UPR.

The UPR is equal to zero if no potential scenarios lead to a result higher than the minimum acceptable performance. In contrast, it equals infinite if all scenarios lead to a value higher than the minimum acceptable performance. Hence, with this metric as objective, variability on the quantity of interest is accepted, as long as the variability results in performances above the minimum acceptable performance (Fig. 3). In addition to the probability of outcomes with better performance, the UPR is affected by how much better that performance is, favoring distributions with large positive outliers. Note that the negative outcomes in the denominator are squared, which adds additional weight to undesirable outcomes. Therefore, the value for a symmetrical distribution is lower than 1: A symmetrical Gaussian distribution with $\Omega$ equal to the mean results in a UPR of 0.56. Note that when the quantity of interest should be minimized (e.g., cost or carbon intensity), downside protection refers to protection against outcomes higher than the desired values, while upside potential refers to the potential of achieving values lower than the desired values. In the formula, the domains to identify beneficial and harmful events are inversed.

**Figure 4:**
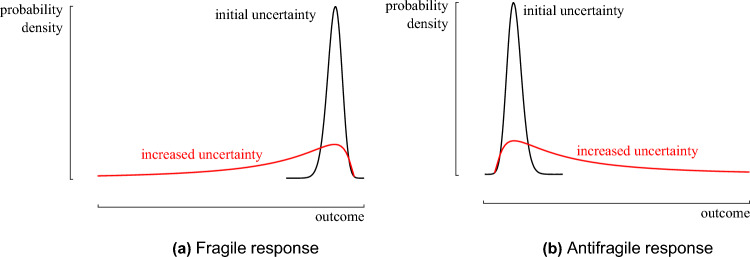
The response of a fragile and antifragile system when the initial uncertainty in the operating environment is underestimated, in the form of a probability density function. Increasing the uncertainty in the random environment augments mostly negative outcomes in the fragile system (**a**). Instead, the antifragile system augments mostly in positive outcomes (**b**). Figure adapted from Taleb^31^.

With the third and final objective, the antifragility of the system is optimized. (Anti)fragility can be identified as the sensitivity of the quantity of interest towards an erroneous estimation of the uncertainty in the random environment^35^. If an increased uncertainty in the random environment augments mainly negative outcomes, the system response is considered fragile (Fig. 4). Instead, the system is considered antifragile if largely positive outcomes are augmented. Such a system is skewed towards positive performances and can be identified by a positive skewness in the distribution of the quantity of interest. The skewness determines the asymmetry that deviates from a symmetrical distribution. Positive skewness indicates that the distribution is skewed to the right (the gain domain), while negative skewness indicates that the distribution is skewed to the left (the loss domain). In conclusion, the skewness identifies if the system has a fragile or antifragile response to misforecasts in a random environment. It circumvents the need for accurately quantifying the probability of (tail) events, as it focuses on optimizing the trend in response of the system towards those events.

The three objectives can be compared with existing optimization strategies for renewable energy systems under uncertainty, such as worst-case scenario optimization and RDO. ADO employs the median to optimize performance in expected conditions, which better represents the central tendency of highly-skewed distributions on the quantity of interest than the mean (used in RDO). The UPR objective minimizes downside variability but allows for upside variability, which mitigates potential overconservatism of RDO and worst-case scenario optimization, where the entire variance is minimized or the design is optimized for the worst-case scenario, respectively. However, the minimum acceptable performance in the UPR remains a subjective choice. Finally, optimizing the skewness addresses the insurmountable challenge of accurately characterizing either the entire uncertainty (in RDO) or the worst-case scenario (in worst-case scenario optimization) on the input parameters, especially for parameters for which limited knowledge is available. Using the skewness as objective provides designs that perform better if the uncertainties on the inputs are higher than anticipated, making it valuable in cases where the uncertainty on the inputs itself is highly uncertain (e.g., the natural gas price in the next decade).

### Multi-objective optimization algorithm

To optimize the objectives, the third version of the Nondominated Sorting Genetic Algorithm (NSGA-III) is adopted^51^. It allows for optimizing complex, non-linear input-output relations without requiring information on the derivative of that relation. The algorithm is a variant of the extensively used NSGA-II framework with improved performance when three or more objectives are present. In short, the algorithm starts from an initial population that contains a set of design samples generated using Latin Hypercube Sampling (LHS)^52^. From the population, offspring are created using binary tournament selection, crossover and mutation rules. The design samples from the population and offspring are organized based on non-dominated sorting, and the top-performing samples are stored in the next population. Out of this new population, offspring are again created, and the procedure is repeated until the termination criterion is reached.

### Framework overview

We developed the ADO framework in Python. Starting from the Python package RHEIA^46^ that performs PCE-assisted RDO, the package has been modified in two main aspects to perform ADO: The NSGA-III optimization method has been integrated from the Python package DEAP^53^, and the specific objectives of the ADO framework were calculated on the distribution generated by the PCEs.

**Figure 5:**
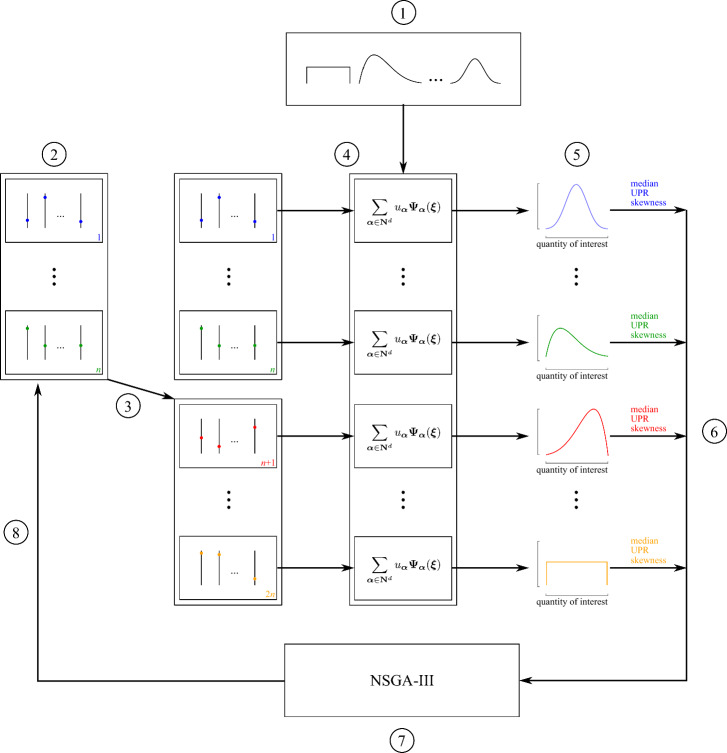
A schematic overview of the Antifragile Design Optimization framework. After characterizing the input parameter distributions (1), an initial population of *n* design samples is defined (2). From this population, *n* offsprings are generated (3). For the 2*n* design samples, PCEs are generated (4) and the distributions on the quantity of interest are calculated (5). From those distributions, the median, Upside Potential Ratio (UPR) and skewness are retrieved (6). The NSGA-III ranks the design samples (7) and updates the initial population with the top-half of the ranked design samples (8).

In summary, the ADO package works as follows (Fig. 5). First, the distributions on the input parameters are characterized (1). After that, an initial set of *n* design samples is created using LHS in the space defined by the bounds on the design variables (2). Out of the initial population, *n* offspring are created using crossover and mutation rules (3). For each design sample in the combined set (initial population + offspring, i.e., 2*n* design samples), a PCE is constructed (4). A Monte Carlo Simulation is performed on each PCE to quantify the distribution of the quantity of interest (5). From each distribution, the objectives (median, UPR and skewness) are quantified (6). In NSGA-III, the design samples are ranked based on their dominance in the objectives (7). The initial population is then updated with the top half of the ranked design samples (8). This updated population (i.e., the next generation) forms the basis for the next offspring. This process is repeated until the computational budget is spent.

To ensure that the PCE surrogate models are accurate during the ADO procedure, the total degree of the polynomials needs to be defined. A fixed total degree is often selected during the entire process of PCE-assisted optimization, as it ensures finalization and tractability of the process^54,55^. To acquire a sufficient total degree for the PCE for each design generated by the ADO method, we applied a screening method^44^. First, a representative set of *n* design samples is generated using LHS. Then, for each design sample, a PCE is constructed based on the truncation scheme for $p=1$. The worst LOO error of all PCEs is collected and compared with a user-defined threshold (generally around 1%^20^). If the worst LOO error is below the threshold, the corresponding total degree can be considered sufficient during the ADO process. If the worst LOO error is above the threshold, the total degree is increased. The process is repeated until the threshold is reached.

## Results

The ADO framework is applied to a renewable energy system case study. In this section, first, the case, the uncertainty characterization of the random environment and the quantity of interest are described. After that, the results are outlined and discussed.

### Case description

**Figure 6:**
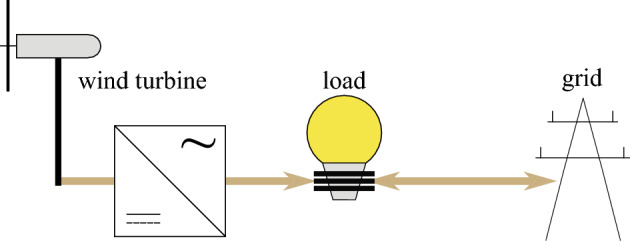
A schematic of the system. The system consists of a community with an electricity demand. The demand is covered by the wind turbine, if sufficient wind power is available at a specific hour of the year. If excess wind power is available, the remaining power is sold to the grid. Instead, when the wind power does not comply with the electricity demand, the remaining demand is covered by the power grid.

**Figure 7:**
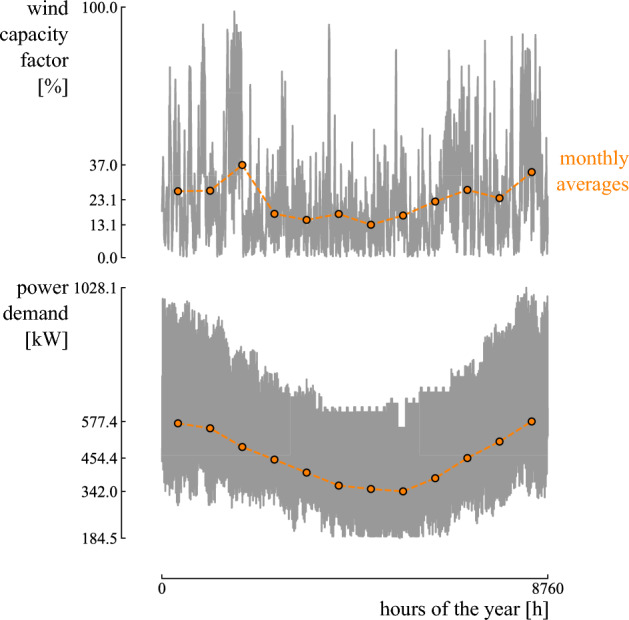
The hourly wind capacity factor and electricity demand profiles considered for the case study.

A grid-connected community is considered in Belgium with an electricity demand of 4 GWh/year. To comply with the demand, installing a wind turbine is considered (Fig. 6). To quantify the performance of the wind turbine, the hourly wind capacity factor for the location of interest is adopted from *renewables.ninja*, based on the performance of a Vestas V66 2 MW wind turbine^56^ (Fig. 7). Hence, for each hour of the year, the community’s electricity demand is (partly) addressed by wind turbine power. If the power provided by the wind turbine is not sufficient, the remaining power is bought from the grid. Instead, if excess wind power is available, the extra power is sold to the grid. The simulation is performed with hourly resolution over one year and extrapolated over the lifetime (25 years). We assume that the compensation for grid reinjection is equal to the wholesale electricity price. Instead, the price for buying electricity is 2.5 times the wholesale electricity price (i.e., the wholesale electricity price corresponds to 40% of the price of purchasing electricity price from the grid, the remaining costs are related to taxes and transmission fees)^57^.

### Uncertainty characterization

**Figure 8:**
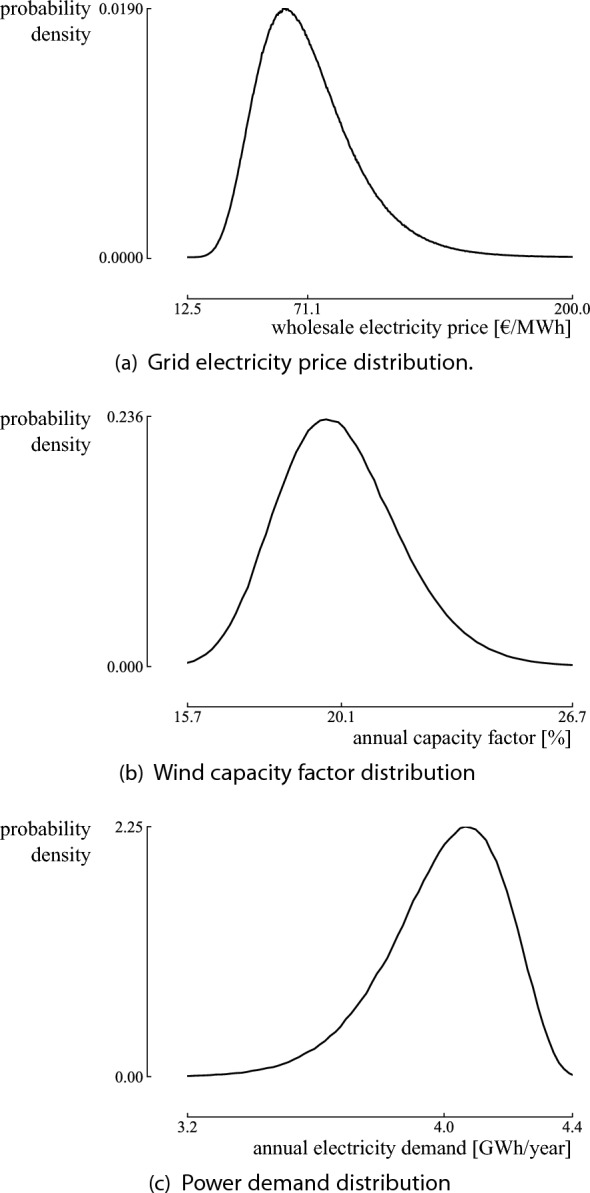
The distributions for the uncertain parameters. The wholesale electricity price and wind capacity factor are defined by right-skewed distributions. The annual electricity demand is defined by a left-skewed distribution, as we assume a higher probability that the annual electricity demand of the household will decrease over the lifetime of the system. The compound uncertainty from these input distributions corresponds to the random environment.

The random environment is defined by three uncertain input parameters: the wholesale electricity price, the wind capacity factor and the power demand (Fig. 8). The PDF of the wholesale electricity price is defined by a lognormal distribution^22^ (Fig. 8a). To define the distribution, quantiles are assigned to the worst case, base case and best case scenarios adopted from the Belgian electricity system operator (see the “Uncertainty characterization” section). The PDF of the annual wind capacity factor is based on historical data and the prediction of the evolution of the capacity factor due to climate change. Based on historical annual capacity factors between 1980 and 2020^56^, the interannual variability is characterized by a normal distribution. A lognormal distribution defines the effect of the future evolution of the capacity factor, based on the expected capacity factors for predicted Shared Socioeconomic Pathways (SSP)^58^: An SSP2-4.5 would increase the onshore wind power energy density in Belgium by 0.2% (30th percentile), while an SSP5-8.5 (highest emission scenario) increases the energy density up to 6% (95th percentile). Summing both distributions results in a lognormal distribution for the annual wind capacity factor (Fig. 8b). Finally, the uncertainty on the annual electricity demand is based on the corresponding range provided by Rixhon et al.^49^. Considering the asymmetric range, a left-skewed Beta distribution is assigned with $P_{10} = 3.75 \text{ GWh } \text{/ } \text{ year }$, $P_{50} = 4 \text{ GWh } \text{/ } \text{ year }$ and $P_{90} = 4.19 \text{ GWh } \text{/ } \text{ year }$ (Fig. 8c).

### Quantity of interest

The Levelized Cost Of Electricity (LCOE) indicates the system cost per unit of electricity demand:

9 $$\begin{aligned} \text{LCOE} = \dfrac{\text{CAPEX}_\text{a} + \text{OPEX}_\text{a} + C_\text{elec,a} }{E_\text{a}}, \end{aligned}$$

where $E_\text{a}$ corresponds to the annualized electricity demand. $\text{CAPEX}_\text{a}$ corresponds to the annualized capital expenses for every system component^59^:

10 $$\begin{aligned} \text{CAPEX}_\text{a} = \text{CRF} \sum _{k=0}^c N_k \text{CAPEX}_k, \end{aligned}$$

where *c* refers to the different system components (wind turbine and converter), and *N* represents the corresponding component size. The Capital Recovery Factor (CRF) is determined by the real discount rate (*i*) and the system lifetime (*L*):

11 $$\begin{aligned} \text{CRF} = \dfrac{i( 1+i )^L}{( 1+i )^L-1}. \end{aligned}$$

The real discount rate considers the effect of inflation (*f*) on the nominal discount rate at the moment of loan ($i'$):

12 $$\begin{aligned} i = \dfrac{i' - f}{1+f}. \end{aligned}$$

In addition to the investment cost, the annualized operating expenses are quantified similarly^59^:

13 $$\begin{aligned} \text{OPEX}_\text{a} = \sum _{k=0}^c N_k \text{OPEX}_k. \end{aligned}$$

Finally, the annualized cost of electricity equals:

14 $$\begin{aligned} C_\text{elec,a} = c_\text{elec} E_\text{a}, \end{aligned}$$

where $c_\text{elec}$ corresponds to the electricity price. The assumptions on the economic parameters are listed in Table 2.

**Table 2:** The cost parameters.

Parameter	Value	Unit	Ref.
CAPEX$_\mathrm {wind~turbine}$	1325	€/kW	^60^
OPEX$_\mathrm {wind~turbine}$	1.1	% of CAPEX	^60^
CAPEX$_\text{converter}$	100	€/kW	^57^
OPEX$_\text{converter}$	3	% of CAPEX	^57^
Discount rate	6	%	^61^
Inflation rate	2	%	^62^

### Results

**Figure 9:**
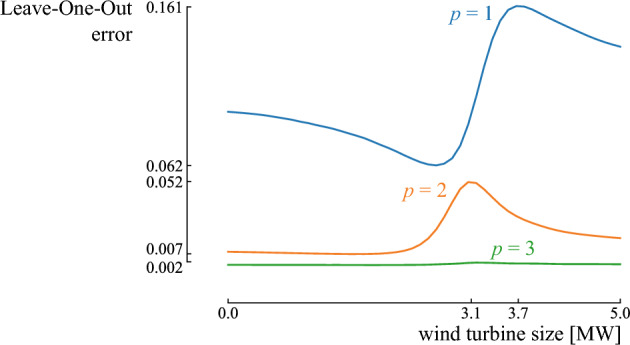
Leave-One-Out error of the Polynomial Chaos Expansion (PCE) for wind turbines ranging from 0 MW to 5 MW, at different maximum total degrees (*p*) of 1, 2, and 3, for each design.

The ADO framework has been applied to optimize the LCOE for the community. The population size (*n*) is set at 30 design samples. Setting the maximum total degree of the PCE model (*p*) to 1, 2, and 3, resulted in a worst-case LOO error of 16.1%, 5.2% and 0.2%, respectively (Fig. 9). Therefore, we adopted a maximum total degree of 3, resulting in a LOO error equal or below 0.2% across the design space. The minimum acceptable performance for the UPR ($\Omega$ in Eq. 8) corresponds to the expected value for the LCOE if the electricity demand is fully covered by grid power (168.1 €/MWh). Hence, this is the expected LCOE when the community continues with the business-as-usual scenario, i.e., it remains entirely dependent on grid electricity.

A Pareto set of optimized designs is achieved, making a trade-off between the three objectives. First, the Pareto front and the characteristics of three notable, optimized designs are discussed. Second, a comparison is provided with the robust design that would have been achieved when using RDO. After that, an illustration of the (anti)fragile character of the designs is presented.

#### Pareto optimized designs

**Figure 10:**
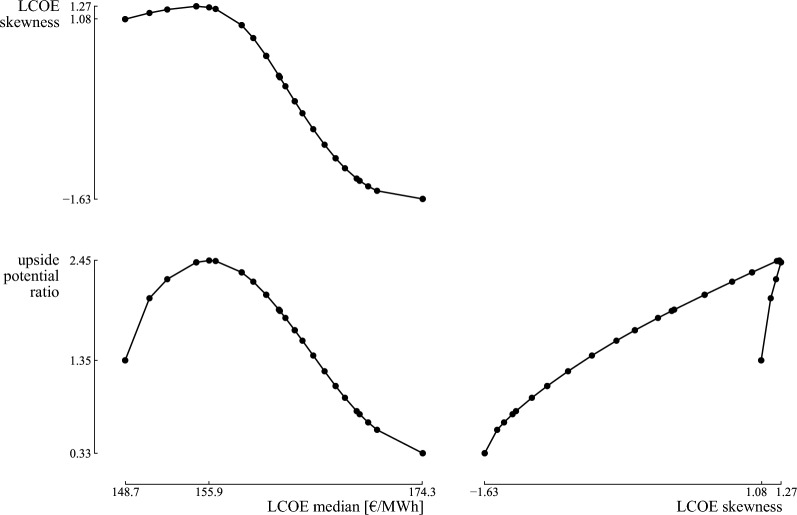
The performance of the Pareto set of optimized designs in the Levelized Cost Of Electricity (LCOE) median, Upside Potential Ratio (UPR) and skewness. A trade-off exists between the three objectives. The optimized median corresponds to a moderate UPR and a relatively bad skewness. The optimized UPR corresponds to the worst skewness, while the optimized skewness corresponds to the worst median.

The results illustrate a trade-off between the three objectives, i.e., the median, UPR and skewness of the LCOE (Fig. 10). The optimized median (148.7 €/MWh) corresponds to a moderate UPR (1.35) and high skewness (1.08). This stochastic performance is realized by installing a 1.4 MW wind turbine. Alternatively, a 2.5 MW wind turbine results in the optimized UPR (2.45) at the expense of an increased LCOE median (155.9 €/MWh) and the worst skewness (1.27) among the optimized designs. The optimized skewness ($-1.63$) corresponds to the worst LCOE median (174.3 €/MWh) and the worst UPR (0.33) among the optimized designs and is realised by a 3.6 MW wind turbine. These three notable designs are discussed in the following paragraphs.

**Figure 11:**
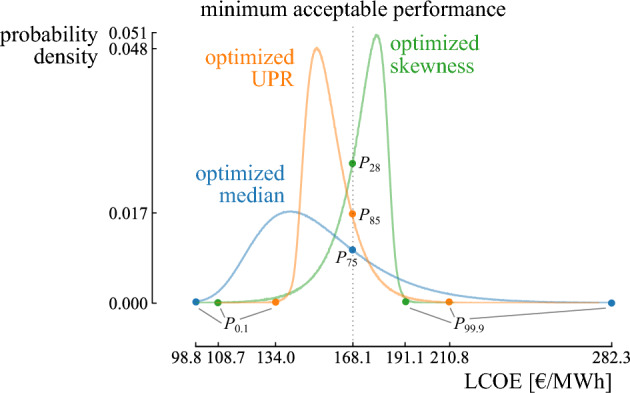
The probability density functions for the designs with optimized median, Upside Potential Ratio (UPR) and skewness. The distributions for the designs with optimized median and optimized UPR are skewed towards negative performances, i.e., an increasing Levelized Cost Of Electricity (LCOE), and thus fragile. The distribution for the design with optimized skewness is skewed towards positive performances, and thus corresponds to an antifragile response.

The first design achieves the optimized LCOE median (148.7 €/MWh) and corresponds to a 1.4 MW wind turbine (Fig. 10). Hence, in expected conditions, this design promises an optimized LCOE. As the median of the distribution is significantly lower than the minimum acceptable value (168.1 €/MWh), the design is likely to return a beneficial LCOE with respect to the random environment. To illustrate, the minimum acceptable value is at the 75th percentile, meaning that the design has a probability of 75% to realize an LCOE below the minimum acceptable LCOE (blue distribution on Fig. 11). This results in a beneficial UPR of 1.35. The positive value for the skewness (1.08) indicates that the design is fragile towards a random environment. The positive skewness is achieved due to the longer tail in the loss domain than in the gain domain. To illustrate, the 99.9th percentile corresponds to 282.3 €/MWh, which is an increase of 133.6 €/MWh (90%) with respect to the median. Instead, the potential decrease in LCOE is limited in the case of a tail event in the gain domain: the 0.1th percentile corresponds to 98.8 €/MWh, which is a reduction of 49.8 €/MWh (34%) with respect to the LCOE median.

The UPR can be improved up to 2.45 by considering a 2.5 MW wind turbine. Hence, this design choice increases the upside potential with respect to the minimum acceptable performance at the expense of increasing the LCOE median up to 155.9 €/MWh and the skewness up to 1.27 (Fig. 10). Notably, despite an increase in the central tendency of the distribution (orange distribution on Fig. 11), the design achieves a higher UPR. This can be explained by a significant reduction in standard deviation (i.e., from 27.0 €/MWh for the design with optimized LCOE median to 10.4 €/MWh for the design with optimized UPR), which significantly decreases the tail in the loss domain. To illustrate, the tail event in the loss domain ($P_{99.9}=$210.8 €/MWh) is reduced by 71.5 €/MWh when compared to $P_{99.9}$ for the distribution with optimized central tendency (282.3 €/MWh). Instead, the change in LCOE in the tail event in the gain domain is less significant, i.e., $P_{0.1} = 134.0$ €/MWh corresponds to an increase of 35.2 €/MWh when compared to $P_{0.1}$ for the distribution of the design with optimized central tendency (98.8 €/MWh). Even though the LCOE median is increased for this design, the median remains below the minimum acceptable performance. Therefore, in addition to shortening the tails, the reduced standard deviation improves the probability of attaining an LCOE below the minimum acceptable value: the minimum acceptable value is at the 85th percentile. Nevertheless, the design remains fragile (skewness $= 1.27$), as the difference between the median and tail event in the loss domain is significantly higher (35%) than the difference between the median and the tail event in the gain domain (14%).

For a wind turbine of 3 MW and larger (LCOE median of 162.5 €/MWh and higher), the skewness has a negative value. Hence, these designs can be considered antifragile. Note that a 3 MW wind turbine corresponds to the design with the lowest LCOE standard deviation, i.e., the robust design. Here, the significance of the uncertainty on the wholesale electricity price is limited, as in expected wind capacity factor conditions, the costs of buying grid electricity and the gains from selling electricity are nearly balanced (i.e., 1 GWh bought and 2.5 GWh sold, which equals the price ratio between buying and selling electricity). For wind turbines with capacities larger than 3 MW, the component of selling electricity gains importance with the rise in wind turbine capacity. Hence, the LCOE standard deviation increases from 8.3 to 11.5 €/MWh. However, in this case, the increasing LCOE variability is mainly present in the gain domain, as a rising wholesale electricity price is now beneficial for the LCOE. Therefore, the skewness decreases to more negative values, leading to distributions with fat tails in the gain domain.

The design with the most negative skewness ($-1.63$), and thus with the most antifragile response, corresponds to a 3.6 MW wind turbine. This design is robust towards variability in the loss domain and benefits from variability in the gain domain. To illustrate, $P_{0.1} =$108.7 €/MWh, which is 65.7 €/MWh (38%) lower than the LCOE median, and $P_{99.9} = 191.1$ €/MWh, which is only 18.8 €/MWh (11%) higher than the LCOE median. However, among the optimized designs, this antifragile design achieves the highest LCOE median (174.3 €/MWh). The high LCOE median results in a relatively low probability of achieving an LCOE below the minimum acceptable value (the minimum acceptable value is at the 28th percentile), and thus a low UPR (0.33).

#### Comparison with robust design and worst-case design

**Figure 12:**
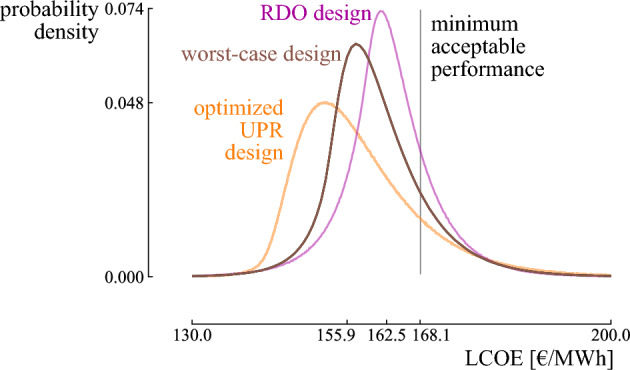
The probability density functions for the design with optimized Upside Potential Ratio (UPR), the robust design from Robust Design Optimization (RDO) and the worst-case design.

To evaluate the new results, we conducted a comparison with existing methods. Specifically, we compared the design with optimized UPR with designs from two other methods: the robust design from RDO that achieves a minimized LCOE standard deviation, and the worst-case design that achieves an optimized LCOE in the worst-case scenario (Fig. 12). While the robust design from RDO can be generated using the same input distributions, the worst-case scenario was determined by tail events from the input distributions: $P_{0.1}$ for the wind capacity factor and $P_{99.9}$ for the energy demand and wind capacity factor.

As the design with optimized UPR is optimized with respect to the minimum acceptable performance, it achieves a higher probability of resulting in an LCOE below this reference (85%) than the robust design from RDO (80%) and the worst-case design (83%). Furthermore, the design with optimized UPR achieves a lower median LCOE (155.9 €/MWh), compared to the robust design from RDO (162.5 €/MWh) and the worst-case design (159.7 €/MWh). Finally, the design with optimized UPR returns a more beneficial LCOE than the robust design from RDO and the worst-case design in 81% and 80% of the possible scenarios, respectively (retrieved from evaluating $10^6$ random samples from the random environment on both designs).

#### Antifragile character

As mentioned in the introduction of this paper, unforeseen events (e.g., war in Ukraine, COVID-19, effects of climate change) lead to unexpected scenarios that significantly affect some input parameters. For example, projections regarding the variability of wind energy as a result of climate change are still subject to a high degree of uncertainty, as has been highlighted in recent research^63^. Consequently, unknown, unforeseen effects can be missed which result in a current underestimation of the wind capacity factor uncertainty. Additionally, scenarios pertaining to future wholesale electricity prices did not consider the global energy crisis of 2022, which has caused extraordinarily high prices^64^.

**Figure 13:**
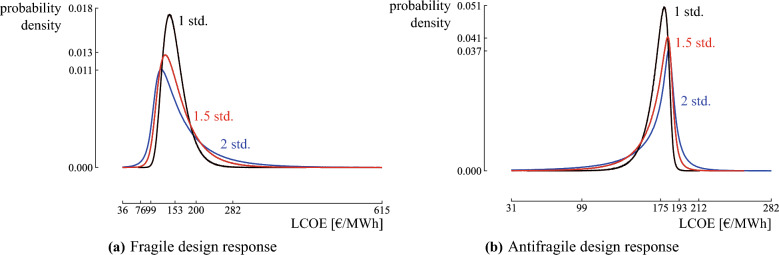
The evolution of the distributions for the fragile and antifragile design when the uncertainty on the input parameters increases. When the standard deviation (std.) is doubled on the input parameters, the fragile design mainly augments outcomes with increased Levelized Cost Of Electricity (LCOE), while the antifragile design mainly augments outcomes with reduced LCOE.

**Figure 14:**
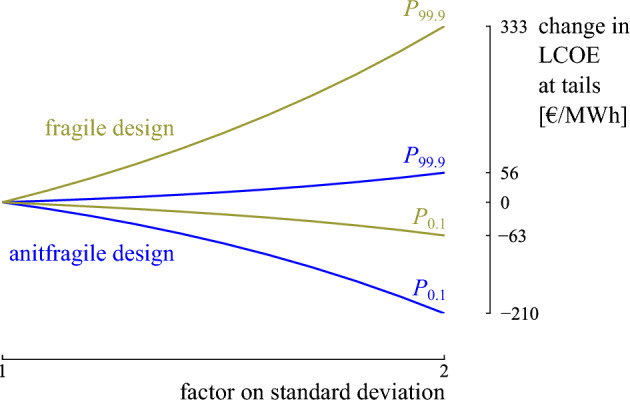
The evolution of the tails at $P_{99.9}$ and $P_{0.1}$ for the fragile and antifragile design for an increasing standard deviation. The evolution of the tails for the fragile and antifragile design illustrates that the antifragile design mainly benefits from increased uncertainty, while the fragile design is mainly harmed.

To illustrate how the designs respond if the uncertainty in the random environment is underestimated, the uncertainty in the random environment is gradually increased, and the corresponding distribution on the LCOE is quantified. Starting from the initial random environment (Fig. 8), the standard deviation on each input parameter distribution is increased by a factor of 2 in steps of 0.1. The distribution on the LCOE is calculated on the design with an optimized tendency (further referred to as *the fragile design*) and the design with optimized skewness (further referred to as *the antifragile design*) for each scenario (Fig. 13). For the fragile design, increasing the uncertainty on the input parameters mainly augments negative outcomes (Fig. 13a). Instead, increasing the uncertainty on the antifragile design mainly augments positive outcomes (Fig. 13b). Hence, the fragile design is subject to accelerating damage under misforecasts, while the antifragile design thrives with accelerating benefits. To clarify, the evolution of the tails at $P_{0.1}$ and $P_{99.9}$ is calculated for varying standard deviation on the input parameters (Fig. 14). For the fragile design, the gain in LCOE at $P_{0.1}$ is only 63 €/MWh when the standard deviation on the input parameters is doubled, while the loss at $P_{99.9}$ is 333 €/MWh. Hence, this design is fragile towards incorrect estimation of the input distributions. Instead, the antifragile design benefits from increased uncertainty: $P_{99.9}$ increases by only 56 €/MWh, while $P_{0.1}$ decreases by 210 €/MWh.

## Discussion

As highlighted in the case study, the three objectives favor different designs that are beneficial in different circumstances. The design with an optimized LCOE median (1.4 MW wind turbine) achieves an optimized LCOE when the most probable scenarios from the input distributions become true, i.e., the median equals 148.7 €/MWh. Although the tail events can nearly double the LCOE with respect to the median, the design provides a probability of 75% that the actual LCOE is better than the minimum acceptable LCOE. A 2.5 MW wind turbine is even more reliable (85%) in terms of resulting in an LCOE below the minimum acceptable LCOE, and the potentially significant losses in the tail are reduced significantly (by 25%). However, the increase in upside potential comes at the expense of an increase in LCOE when the most probable scenarios become true: the median increases up to 155.9 €/MWh (5%). Nevertheless, both designs are fragile towards underestimating the random environment: the LCOE significantly increases for an increasingly uncertain random environment (e.g., the LCOE in the tail for the 1.4 MW wind turbine grows up to 3.3 times higher than the median when the uncertainty in the random environment is doubled). Instead, a 3.6 MW wind turbine is antifragile towards an increasingly uncertain random environment, reducing the LCOE with 120% in the tail when the input uncertainty is doubled. The antifragile characteristic comes at the expense of an increase of 17% in the median, making this design less beneficial in the most probable scenarios. However, this expense is based on an accurate representation of the random environment, which is not validatable. Even more, it likely underestimates the uncertainty in the real world. Therefore, adopting the antifragile design is a measure of insurance towards underestimating the random environment at the expense of an increase in LCOE by 17% in the expected conditions.

The selection of a design over the others depends on certain conditions. The design with optimized median is preferred when the uncertainty characterization of the input parameters is reliable (e.g., based on a significant amount of quality data) and the resulting uncertainty on the quantity of interest is low (e.g., a coefficient of variation ($\sigma / \mu$) below $0.25\%$ on the performance was considered acceptable by Giorgetti et al.^65^). With a low variability on the output—based on reliable inputs—the performance is unlikely to vary significantly from the expected performance during the lifetime of the system. Instead, if the variability on the quantity of interest results in a significant violation of minimum acceptable performance, the alternative designs with optimized UPR are preferable. Finally, designs with an optimized skewness are beneficial when—despite best efforts—the uncertainty characterization is unreliable, simply because the future evolution of some input parameters is impossible to predict and their uncertainty is likely underestimated (e.g., the future evolution of the natural gas price). In this case, the variability on the quantity of interest might be significantly underestimated, leading to an overestimated probability of operating in expected conditions and an underestimation of violating the minimum acceptable performance. For the case study, due to the likelihood of underestimating the uncertainty of the grid electricity price, we suggest adopting a design with negative skewness from the Pareto front (Fig. 10), at the expense of a reduced UPR and increased mean. In the case of an higher-than-predicted uncertainty on the input parameters, both the mean and the UPR will improve (as the probability to operate in the gain domain will increase), while, for instance, the design with optimized median will significantly worsen in performance when the uncertainty on the inputs was underestimated (as presented in the “Antifragile character” section). It is important to note that while this decision-making process offers guidance, the final choice ultimately depends on the user’s risk aversion.

The method has two main limitations. First, the UPR is sensitive to the choice of the minimum acceptable performance. As it is a user-defined constant, the optimization results are user-specific and adapted to the personal reference, which makes it difficult to extrapolate the results on the UPR towards similar systems with, e.g., a different level of risk aversion. Second, the method remains significantly driven by the initial assumption of the input distributions, even though the skewness circumvents the underestimation of the random environment. To address this issue, capturing the uncertainty on the input distributions by imprecise probabilities (probability boxes) can be investigated^66^. Despite the complicated interpretation of the results—the outputs become imprecise as well—several random environments can be propagated and considered during the optimization procedure^67^.

The ADO method can be easily applied to any other system model in any other field. The system model is considered a black box as the UQ and optimization methods are non-intrusive. Hence, the method generates samples for UQ and optimization purposes and evaluates the deterministic response of the system model. Therefore, the nature of the system model is irrelevant as long as the quantity of interest is continuous.

## Conclusion

In this paper, we revisited the objectives for optimization under uncertainty in a probabilistic framework applied to renewable energy systems. As skewed distributions define the random environment of energy systems and the simulation models are non-linear—they cover a significant period (years) with high temporal resolution (hours)—the central tendency of the distribution on the quantity of interest is estimated by the median. The Upside Potential Ratio is adopted to optimise the variability, as it penalizes downside variation and encourages upside variation. Finally, the skewness is adopted as an objective to overcome the inevitable underestimation of the uncertainty on critical input parameters.

The methodology has been applied to the design of a wind turbine for a community. The design with an optimized LCOE median (1.4 MW wind turbine) achieves an optimized LCOE when the most probable scenarios for the input scenarios become true. It promises an appealing potential to perform better than the minimum acceptable performance (in 75% of the scenarios). A 2.5 MW wind turbine is a valid alternative to the 1.4 MW wind turbine, as it increases the probability of performing better than the minimum acceptable LCOE by $10 \%_{\text{ abs }}$ and significantly reduces the impact of tail events in the loss domain (25%), at the expense of a slight increase in performance when the random environment performs near the most probable scenarios (5%). Moreover, it achieves a more appealing performance than the conventional robust design, i.e., it achieves a lower LCOE median (4%) and performs better than the robust design in 81% of the scenarios. Nevertheless, both designs are fragile towards underestimating the random environment. A 3.6 MW wind turbine is antifragile, reducing the LCOE with 120% in the tail when the uncertainty in the random environment is higher than expected. The antifragile characteristic comes at the expense of an increase of 17% in the median, making this design less beneficial in the expected scenarios.

The main limitation is the subjective decision on the minimum acceptable performance to determine whether the variation is beneficial or harmful. Although essential with respect to the risk profile of the decision-maker, the subjective minimum acceptable performance significantly determines the optimization results, which limits the extrapolation of the results to other similar systems.

Even though the skewness indicates the antifragile character with respect to an erroneous estimation of the random environment, the optimization process is significantly driven by the initial distributions assumed on the input parameters. Therefore, in future works, we will introduce imprecise probabilities to propagate several random environments simultaneously through the system model^67^, such that the different random environments are considered during the optimization procedure.

## Data Availability

The data and materials are available from the corresponding author upon reasonable request.
